# A Bitter Taste in Your Heart

**DOI:** 10.3389/fphys.2020.00431

**Published:** 2020-05-08

**Authors:** Conor J. Bloxham, Simon R. Foster, Walter G. Thomas

**Affiliations:** ^1^School of Biomedical Sciences, Faculty of Medicine, The University of Queensland, St Lucia, QLD, Australia; ^2^Department of Biochemistry and Molecular Biology, Monash Biomedicine Discovery Institute, Monash University, Clayton, VIC, Australia

**Keywords:** taste receptors, G protein-coupled receptors, cardiac physiology, signaling, polymorphisms, bitter ligands

## Abstract

The human genome contains ∼29 bitter taste receptors (T2Rs), which are responsible for detecting thousands of bitter ligands, including toxic and aversive compounds. This sentinel function varies between individuals and is underpinned by naturally occurring T2R polymorphisms, which have also been associated with disease. Recent studies have reported the expression of T2Rs and their downstream signaling components within non-gustatory tissues, including the heart. Though the precise role of T2Rs in the heart remains unclear, evidence points toward a role in cardiac contractility and overall vascular tone. In this review, we summarize the extra-oral expression of T2Rs, focusing on evidence for expression in heart; we speculate on the range of potential ligands that may activate them; we define the possible signaling pathways they activate; and we argue that their discovery in heart predicts an, as yet, unappreciated cardiac physiology.

## Extra-Oral Expression of Bitter T2Rs

*TAS2R*/T2Rs (*gene* and protein) were first discovered within type II taste receptor cells in the tongue and act as sentinels in protecting against the ingestion of potentially toxic substances (Chandrashekar et al., 2000; Lu et al., 2017). Since these pioneering studies, T2R expression has been reported in a multitude of extra-oral tissues, including the gut, lungs, brain, and heart (Shah et al., 2009; Foster et al., 2013; Garcia-Esparcia et al., 2013), but their complete function(s) in physiology and pathophysiology remain to be defined. In Table 1, we have summarized the location, expression profile and proposed function for the T2R family across a range of human tissues and cells. In regard to function, we would offer a note of caution that a number of studies (listed in Table 1) have proposed functions based on stimulation with various bitter compounds in the micromolar to millimolar range where the selectivity and specificity toward T2Rs may reasonably be questioned. Despite this, the expression of T2Rs within the cardiovascular system, particularly the heart and vasculature, has gained significant interest in recent years. Following our initial discovery of *TAS2Rs* within the heart (Foster et al., 2013), a number of subsequent studies have focused on the vasculature (Lund et al., 2013; Manson et al., 2014; Upadhyaya et al., 2014; Chen et al., 2017). An unambiguous definition of their function has, however, lagged behind the capacity to demonstrate their expression.

**TABLE 1:** Distribution, expression profile, proposed function, and technique used for the detection of extra-oral *TAS2R*/T2R expression.

**System**	**Cell/tissue**	**TAS2Rs**	**Proposed function**	**Technique(s) used**	**Year**
Cardiovascular	Heart	*TAS2Rs (TAS2R3, 4, 5, 9, 10, 13, 14, 19, 20, 30, 31, 43, 45, 46, 50)*	Nutrient sensing, contractility	RT-qPCR	Foster et al. (2013)
	VSMCs	*TAS2R46, T2R46*	Vasodilation	RT-qPCR, IHC, Ca^2+^ imaging	Lund et al. (2013)
	Artery (pulmonary)	*TAS2Rs (TAS2R3, 4, 10, 14)*	Vasodilation	PCR	Manson et al. (2014)
	Pulmonary VSMCs	*TAS2Rs (TAS2R1, 3, 4, 5, 7, 8, 9, 10, 13, 14, 19, 20, 30, 31, 39, 42, 43, 45, 46, 50, 60*	Vasoconstriction	RT-qPCR	Upadhyaya et al. (2014)
	Artery (omental)	*TAS2Rs (TAS2R3, 4, 7, 10, 14, 39, 40), T2R7*	Vasodilation	RT-qPCR, WB	Chen et al. (2017)
	Venous blood	*TAS2R38*	Sensing biofilm formation	RT-qPCR	Cantone et al. (2018)
Endocrine	Adipocyte	*TAS2R46*	–	RT-qPCR	Lund et al. (2013)
	Thyroid, Nthy-ori 3-1 cells	*TAS2Rs (TAS2R3, 4, 5, 9, 10, 13, 14, 16, 20, 30, 38, 42, 43, 45, 46)*	Modulation of thyroid hormone production	RT-qPCR	Clark et al. (2015)
	Pancreas (ductal adenocarcinoma biopsy), SU8686 cells, T3M4 cells, MiaPaCa-2 cells, RLT cells	T2R38	Cancer drug resistance, microbiota link to cancer	Cytofluorometry, WB	Gaida et al. (2016b)
	PANC-1 cells, BxPC-3 cells	T2R10	Cancer drug resistance	Flow cytometry	Stern et al. (2018)
Gastrointestinal	Colon, Gut HuTu 80 cells, NCI-H716 cells	*TAS2Rs (TAS2R3, 4, 5, 10, 13, 20, 30, 31, 38, 39, 40, 42, 43, 45, 46, 50, 60)*	–	RT-PCR, Ca^2+^ imaging	Rozengurt et al. (2006)
	Cecum, NCI-H716 cells	*TAS2R9*	Glucose homeostasis	RT-PCR, Ca2^+^ imaging	Dotson (2008)
	Colon	*TAS2R (TAS2R1, 4, 38)*	–	RT-PCR	Kaji et al. (2009)
	Caco-2 cells	*TAS2R38*	Limits absorption of bitter tasting/toxic substances	RT-PCR, siRNA	Jeon et al. (2011)
	Saliva	*TAS2R43*	Balkan endemic nephropathy (BEN)	RT-PCR	Wooding et al. (2012)
	HuH7 cells	T2R38	–	Cytofluorometry	Gaida et al. (2016b)
	Parotid gland	*TAS2R38*	–	IHC	Wolfle et al. (2016)
	Stomach (epithelial and parietal cells), HGT-1 cells	*TAS2Rs (TAS2R1, 3, 4, 5, 7, 9, 10, 13, 14, 16, 19, 20, 30, 31, 38, 39, 40, 41, 42, 43, 46, 50)*	Gastric acid secretion	RT-PCR	Liszt et al. (2017)
Integumentary	MDA-MB-231 cells, MCF-7, MCF-10 cells	*TAS2R (TAS2R1, 4, 10, 20, 38), T2R38*	–	qPCR, flow cytometry, Ca^2+^ mobilization	Singh et al. (2014)
	HPKs, HeCaT cells	*TAS2R1, TAS2R38, T2R1, T2R38*	Keratinocyte differentiation	IHC, RT-PCR	Wolfle et al. (2016)
	Skin biopsies with epidermis and dermis	*TAS2Rs (TAS2R3, 4, 5, 9, 10, 13, 14, 16, 19, 20, 30, 31, 38, 39, 40, 41, 42, 43, 45, 46, 50, 60)*	–	qPCR	Shaw et al. (2018)
Immune	Leukocytes, blood T- and B-lymphocytes, monocytes, neutrophils, NK cells	*TAS2Rs (TAS2R4, 5, 10, 13, 14, 19, 20, 45, 46, 50)*	Anti-inflammatory role in asthma	Microarray, RT-qPCR, cytokine ELISA	Orsmark-Pietras et al. (2013)
	Core blood-derived mast cells, HMC1.2 cells	*TAS2Rs (TAS2R3, 4, 5, 10, 13, 14, 19, 20, 46)*	Anti-inflammatory	RT-qPCR	Ekoff et al. (2014)
	Neutrophils, monocytes, HL-60 cells and U937 cells	T2R38	Sensing biofilms	Cytofluorometry and laser scan microscopy	Gaida et al. (2016a)
	Lymphocytes	T2R38	Immune modulation	Flow cytometry	Tran et al. (2018)
Skeletomuscular	BMSCs, osteocyte, chondrocyte	*TAS2R46, T2R46*	Chemosensory	iTRAQ-based mass spectrometry, RT-qPCR, flow cytometry, IHC, Ca^2+^ imaging	Lund et al. (2013)
Nervous	Frontal cortex	*TAS2Rs (TAS2R5, 10, 13, 50)*	–	RT-qPCR, microarray	Garcia-Esparcia et al. (2013)
	Cortex (pyramidal cells), cerebellum (Purkinje cells), hippocampus, SH-SY5Y cells	*TAS2R16, T2R16*	Neurite growth	IHC, PCR	Wolfle et al. (2015)
	Dorsolateral prefrontal cortex	*TAS2Rs (TAS2R4, 5, 10, 13, 14, 50)*	Cognition	RT-qPCR	Ansoleaga et al. (2015)
	Purkinje cells	T2R38	–	IHC	Wolfle et al. (2016)
	Neurons/glial cells	T2R38	–	IHC	Wolfle et al. (2016)
Urogenital	Bladder cancer biopsies	*TAS2R1*	–	FISH	Zheng et al. (2004)
	Testis	*TAS2Rs (TAS2R14, 16, 38)*	–	RT-qPCR	Behrens et al. (2006)
	HeLa cells, DU145 cells	*TAS2R13*	Cytokinesis	RT-PCR, siRNA screen	Zhang et al. (2012)
	SKOV-3 cells	T2R38	–	Cytofluorometry, WB	Gaida et al. (2016b)
	Placenta, JEG-3 cells	T2R38	–	IHC, immunofluorescence, restriction enzyme-based detection, Ca^2+^ mobilization	Wolfle et al. (2016)
	Kidney	T2R38	–	IHC	Wolfle et al. (2016)
	Cervix	T2R38	–	IHC	Wolfle et al. (2016)
	Myometrium, hTERT-HM cells	*TAS2R (TAS2R5, 10, 13, 14); TAS2R (TAS2R3, 4, 5, 7, 8, 10, 13, 14, 31, 39, 42, 43, 45, 50)*	–	IHC, Ca^2+^ mobilization, RT-PCR	Zheng et al. (2017)
	Sperm	*TAS2R14, TAS2R43*	Sperm motility and maturation	Allele-specific PCR	Gentiluomo et al. (2017)
	Ovarian cystadenocarcinoma tumor, uterine tissue, OVCAR4 cells, OVCAR8 cells, SKOV3 cells, IGROV1 cells, HEC-1a cells, BPH1 cells, PC3 cells, LNCAP cells, DU145 cells	*TAS2R (TAS2R1, 4, 10, 14, 38)*	Cell survival	qPCR, siRNA screen, WB	Martin et al. (2018)
Respiratory	Bronchial epithelial cells	*TAS2Rs (TAS2R1, 3, 4, 7, 8, 9, 10, 13, 14), T2R4, T2R43, T2R46*	Motile cilia clearance of inhaled pathogens	Microarray, RT-PCR, IHC, Ca^2+^ imaging, ciliary beat frequency assay	Shah et al. (2009)
	ASM, trachea	*TAS2Rs (TAS2R1, 3, 4, 5, 8, 9, 10, 13, 14, 19, 20, 30, 31, 42, 45, 46, 50)*	Relaxation of isolated ASM, bronchodilation	RT-qPCR, Ca^2+^ imaging, isolated trachea, single cell mechanics/membrane potentials	Deshpande et al. (2010)
	16HBE cells	*TAS2R38, TAS2R46*	–	RT-PCR, Ca^2+^ mobilization, cAMP accumulation	Cohen et al. (2012)
	Upper respiratory epithelium	*TAS2R38, T2R38*	NO-mediated increase in ciliary beat frequency/mucous clearance and antibacterial effects in respiratory infection	IHC, Ca^2+^ imaging, NO production, ciliary beat frequency assay, mucous clearance assay, bactericidal assay	Lee et al. (2012)
	Nasal epithelial cells	*TAS2R38*	Innate immunity	RT-qPCR	Lee et al. (2012)
	Solitary or brush chemosensory cells	*TAS2R (TAS2R4, 14, 46)*	Innate immunity	RT-qPCR	Barham et al. (2013)
	Bronchi	*TAS2Rs (TAS2R3, 4, 5, 7, 8, 9, 10, 14, 19, 20, 31, 38, 39, 43, 45, 46)*	Bronchodilation	RT-qPCR, organ bath	Grassin-Delyle et al. (2013)
	Alveolar macrophages	*TAS2Rs (TAS2R3, 4, 5, 7, 8, 9, 10, 14, 19, 20, 31, 38, 39, 43, 45, 46)*	–	RT-qPCR	Grassin Delyle et al. (2014)
	CuFi-1 cells, NuLi-1 cells	*TAS2Rs (TAS2R3, 4, 5, 8, 9, 10, 13, 14, 19, 20, 30, 31, 43, 45, 46, 50, 60)*	–	nCounter, flow cytometry	Jaggupilli et al. (2017)

The expression of *TAS2R*s in different tissues and cell lines has been examined using RT-PCR, qPCR, microarray techniques as well as RNAseq (Flegel et al., 2013). Most recently, Jaggupilli et al. (2017) used nCounter gene expression analysis to characterize the expression of the 29 human *TAS2Rs* in a variety of cell lines (Table 1). Their results showed that *TAS2R14* and *TAS2R20* were highly expressed; *TAS2R3, −4, −5, −10, −13, −19*, and *−50* were moderately expressed; *TAS2R8, −9, −21* and *−60* had low level of expression; and *TAS2R7, −16, −38, −39, −40, −41*, and *−42* were barely detectable. The nCounter technique relies on hybridization of complementary probes (spanning 100 nucleotide bases) for each gene, and hence, *TAS2R30, −31, −43, −45*, and −*46* could not be accurately discerned from one another, as they share >92% homology. Nevertheless, this data clearly shows that some T2Rs are broadly and differentially expressed, whereas others are more restricted in their tissue distribution.

## Model Systems for Expressing T2Rs and Defining Their Function

In attempting to define the function of, and to identify ligands for, the T2Rs, researchers have established heterologous expression systems in human cells (e.g., HEK293 or HEK293T) (Meyerhof et al., 2010). However, the use of these cells for understanding the underlying mechanisms and signaling pathways within cardiovascular tissues/cells has obvious limitations. Firstly, due to the insufficient cell surface targeting of T2Rs in heterologous cells (Chandrashekar et al., 2000), chimeric T2Rs encompassing the amino terminus of the rat somatostatin receptor subtype 3 are often used to improve expression and functionality (Bufe et al., 2002; Behrens et al., 2006). Furthermore, a chimeric G protein consisting of the Gα_16_ and 44 amino acids of gustducin attached to the carboxyl terminus is widely used in calcium mobilization assays (Liu et al., 2003; Ueda et al., 2003). Gα_16_ has been coined the ‘universal adaptor’ due to its ability to interact with numerous GPCRs and provides a robust readout for receptor activation, including for T2Rs (Ueda et al., 2003). While these artificial heterologous systems have proven useful in identifying ligands for orphan receptors (Meyerhof et al., 2010) and interrogating the structure-function aspects of T2Rs (Brockhoff et al., 2010), the field is now moving toward more relevant cellular models with endogenous receptors and signaling partners (Freund et al., 2018).

Studies using the aforementioned heterologous expression system have demonstrated that the majority of T2Rs form oligomers, both homodimers and heterodimers (Kuhn et al., 2010). However, unlike the situation for umami/sweet taste sensation (requiring dimerization of T1R1/T1R2 and T1R1/T1R3), T2R homodimers did not appear to alter the pharmacology of the receptors, nor do they have obvious influence on protein expression or membrane localization (Kuhn et al., 2010). In contrast, Kim et al. (2016) used immuno-fluorescent microscopy to show that the co-expression of the adrenergic (ADRβ_2_) receptor with T2R14 resulted in a ∼3-fold increase in cell-surface expression of T2R14. Co-immunoprecipitation and biomolecular fluorescence complementation experiments confirmed that the increase of cell-surface expression was attributed to the formation of T2R14:ADRβ_2_ heterodimers. These complexes may be particularly important in heart where the actions of adrenergic receptors are well described. Interestingly, co-immunoprecipitation and co-internalization of ADRβ_2_:M71 OR (mouse 71 olfactory receptor) was observed in response to their specific ligands (Hague et al., 2004). These seminal observations in heterologous systems need to be confirmed and extended with endogenous models to clarify our understanding of how T2Rs function *in vivo* and to define their potential modulation of (or by) established GPCRs.

Another important issue in considering model expression systems for studying T2Rs is the requirement for appropriate accessory proteins and correct post-translational processing. It is now well-established that chemosensory receptors [e.g., odorant (McClintock et al., 1997) and pheromone (Loconto et al., 2003) receptors] rely on endogenous proteins in order to be targeted to the cell-surface. A study by Behrens et al. (2006) demonstrated that certain members of the receptor-transporting protein (RTP) and receptor expression enhancing protein (REEP) families enhance cell-surface localization and functionality of certain *TAS2Rs*, likely through protein–protein interactions. Furthermore, it was shown that varying combinations of these proteins are expressed endogenously within tissues (circumvallate papillae and testis) that express *TAS2R* genes. Interestingly, the human heart differentially expresses REEP 1, 2, 3, 5, and 6 across heart regions (Doll et al., 2017), suggesting that efficient cell-surface *TAS2R* expression may also be region specific. Nonetheless, these trafficking proteins do not universally promote T2R functionality, for instance T2R14 showed no increase in capacity to mobilize calcium when co-expressed with either RTP or REEP (Behrens et al., 2006). There is accumulating evidence that the degree of T2R membrane insertion is dependent on the specific tissue. As T2Rs are detected in a myriad of tissues, multiple endogenous mechanisms may contribute to their appropriate expression and localization. As for many GPCRs, *N*-glycosylation of T2Rs is important for cell-surface localization–Reichling et al. (2008) reported that glycosylation of the second extracellular loop is essential for the recruitment (via association with the cellular chaperone calnexin) and insertion of *TAS2R*s in the cell membrane; moreover, the function of non-glycosylated *TAS2R*16 could be rescued when co-expressed with RTP3 and RTP4.

## The Cardiac Gpcr Repertoire Includes T2Rs

The human heart expresses over 200 different GPCRs (Wang et al., 2018), some of which are critical for regulating cardiac morphology and function (Capote et al., 2015). Intriguingly, the gene transcripts for more than half of the *TAS2R* family were detected in both left ventricle and right atria (Foster et al., 2013) ranging in abundance between that observed for two classically important cardiac GPCRs – the angiotensin II type 1 receptor and β_1_-adrenergic receptor (ADRβ_1_). It is notable that the expression of *TAS2R14* was equivalent to that of ADRβ_1_ in the left ventricle. These findings are supported by publicly available Illumina Human BodyMap 2.0 project RNA-seq dataset (Flegel et al., 2013), which showed widespread *TAS2R* expression in human tissues and highest expression of *TAS2R14* in heart. It is important, however, to note that T2Rs are not uniformly detected by all techniques, with *TAS2R9*, *TAS2R39*, and *TAS2R45* not detected in the Illumina RNA-seq data set, but detected by qPCR (Foster et al., 2013). These differences could reflect individual variations, noting the body map is from one patient or the more specific nature of RNA-seq over qPCR. Interestingly, the expression of *TAS2Rs* are differentially regulated with age in mice (Foster et al., 2013), but not with sex or in heart failure (Foster et al., 2015a). Furthermore, analysis of the publicly available GTEx LDACC and BioGPS Human Cell Type and Tissue Gene Expression Profiles RNA-seq datasets, highlight the expression of GNAT3 (the taste receptor specific G protein, Gα_Gustducin_) in a variety of human tissues, including the heart.

We previously investigated the factors contributing to cardiac *TAS2R* gene expression *in silico* (Foster et al., 2015a). Similar to rodent *Tas2rs*, there was no evidence of enrichment for particular transcription factor binding sites in the proximal promoter regions of the human *TAS2R* genes. However, we observed that*TAS2R14* (the most abundantly expressed) had the strongest evidence of regulatory activity in its promoter region, i.e., active methylation marks overlapping with the DNase I hypersensitivity cluster. On this basis, although we cannot rule out the presence of specific transcription factors that regulate TAS2R gene expression, we reason that the proximal regulatory regions for some, but not all, *TAS2R* genes might show a basal level of transcriptional activity. This, combined with their multigene cluster expression profiles could facilitate preferential transcription of the specific *TAS2Rs* (Foster et al., 2015a).

The heart is made up of 2–3 billion cardiomyocytes and yet these cells constitute less than a third of all heart tissue (Tirziu et al., 2010). The remaining, more than two thirds of the heart consists of smooth muscle, fibroblasts, other connective tissue cells, endothelial cells, sinoatrial cells, atrioventricular cells, Purkinje cells, pluripotent cardiac stem cells, mast cells, and other immune system-related cells (Tirziu et al., 2010). We have demonstrated that certain *Tas2rs* (rodent) were expressed within both cardiomyocytes and fibroblasts, as well as their downstream signaling effectors (*Gnat3*, *Plcβ2*, *Trpm5*) (Foster et al., 2013). These data suggest that specific cells within the heart may express varying populations of *TAS2R*, similar to that seen in other systems (Table 1). As technology advances, including single cell sequencing and proteomics (Uhlen et al., 2015), the topography of T2Rs within the heart will provide insight into how these receptors function within this system.

## Signaling and Function of T2Rs Within the Cardiovascular System

The binding of bitter ligands to T2Rs results in a conformation change in the receptor allowing it to interact with Gα_Gustducin_ and Gβ_1/3_γ_13_ (Huang et al., 1999), which then activate subsequent downstream pathways (Yan et al., 2001). Knockout (KO) studies have provided conclusive evidence supporting these signaling pathways. Mice lacking either PLCβ2 or TRPM5 exhibited diminished or ablated taste responses to bitter compounds (Zhang et al., 2003). Furthermore, Gα_Gustducin_ KO mice had increased levels of cAMP, compared to the wild-type mice as well as displaying severely impaired responses to the tested compounds (Clapp et al., 2008). As with Gαi-family G proteins, Gα_Gustducin_ can decrease the levels of cAMP, via the activation of phosphodiesterases, which has been observed in response to two bitter compounds, denatonium and strychnine (Yan et al., 2001). Finally, mice that were genetically modified to express novel human T2Rs demonstrated a strong aversive response to ligands that was not evident in wild-type mice (Mueller et al., 2005).

With the discovery of *TAS2R* expression in cardiac tissue (Foster et al., 2013), defining the signaling transduction pathway is of particular interest, yet there is limited evidence for the presence of all the classical taste signaling components in heart. The expression of Gα_Gustducin_ has been shown in human heart tissue, and is particularly enriched in cardiomyocytes (BioGPS Human Cell Type and Tissue Gene Expression Profiles RNA-seq datasets). However, studies have not observed Gγ_13_ (Huang et al., 1999) or TRPM5 (Demir et al., 2014). TRPM4 is present within human heart tissues (Guinamard et al., 2004; Demir et al., 2014), however, both TRPM4 and TRMP5 are considered necessary for taste signal transduction (Dutta Banik et al., 2018). Hence, alternative signal transduction pathways that could mediate the effects of taste receptors in the cardiovascular system should be considered.

**FIGURE 1:**
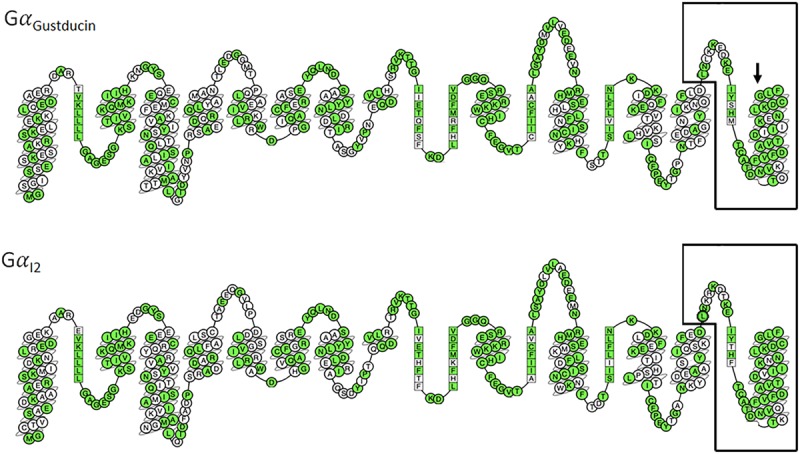
Comparison of 2D snake representations of Gα_Gustducin_ and Gα_i2_ [constructed via GPCRdb (Isberg et al., 2016)]. Amino acids are labeled following the Ballesteros–Weinstein numbering system. Conserved amino acids are colored green. The final 44 amino acids are known to be necessary for T2R coupling and signaling (Ueda et al., 2003) and are highlighted by black boxes. Arrow points to the amino acid (Glycine^352^) in Gα_Gustducin_ that disrupts T2R coupling when mutated to proline (Ruiz-Avila et al., 2001).

A study by Ueda et al. (2003) demonstrated that T2R16 could couple to a chimeric G protein consisting of the N-terminus of Gα_16_ and the last 44 amino acids of either Gα_Gustducin_, Gα_t2_or Gα_i2_. Furthermore, the expression of all three of these Gα subunits have been identified in taste receptor cells, with the frequency of Gα_i2_ being higher than that of Gα_Gustducin_ (Ueda et al., 2003). Figure 1 shows a comparison of Gα_Gustducin_ and Gα_i2_, highlighting the highly conserved amino acid residues and the region known to interact with *TAS2R*s. Substitution of glycine^352^ for proline in Gα_Gustducin_ disrupts T2R interaction with Gα_Gustducin_, although its coupling to the Gβγ and effector molecules was preserved (Ruiz-Avila et al., 2001). This suggests that the extreme C terminus of both Gα_Gustducin_ and Gα_i2_ are capable of, and necessary for T2R:G protein coupling and transduction. Importantly, in human airway smooth muscle (HASM), the reported expression of Gα_i2_ expression was 100-fold higher than that of Gα_Gustducin_ and T2R14 was shown to couple to all Gα_i_ proteins, particularly Gα_i2_ (Kim et al., 2017). The use of pertussis toxin was able to abrogate the T2R mediated relaxation in HASM (Kim et al., 2017), consistent with previous studies, where T2Rs has been shown to couple with inhibitory signaling pathways (Ozeck et al., 2004). The actions of T2R may also include other inhibitory type processes, such as described by Zhang et al. (2013) in airway smooth muscle cells (Lu et al., 2017). Taken together these observations suggest that depending on the level of G protein expression and the strength of the subsequent signal, T2Rs likely couple and signal in a cell/tissue specific manner, which may include (or not) Gα_Gustducin_.

**FIGURE 2:**
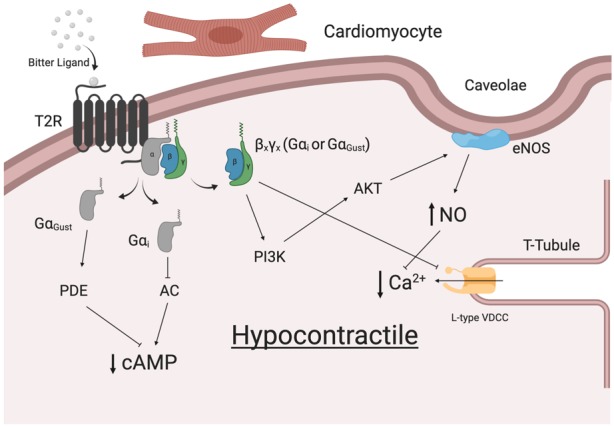
Potential signaling pathway for T2Rs in human cardiomyocytes.

Indeed, T2R signaling within cardiac cells might reasonably reflect those described for the respiratory system and vascular systems (summarized in Figure 2). The heart is known to express specific varying combinations of Gα (including Gα_i2_), Gβγ and various signaling effector molecules (Doll et al., 2017). In a series of experiments in Langendorff-perfused mouse hearts, we observed dose-dependent negative inotropic effects in response to bitter ligands (Foster et al., 2014). A ∼40% decrease in left ventricular developed pressure and an increase in aortic pressure in response to sodium thiocynate were shown to be Gα_i_-dependent. Some alterations in cardiovascular physiology were not attributed to G proteins (not blocked by pertussis toxin and gallein), however, it was shown that rodents express GNAT3 (Gα_Gustducin_) in their cardiomyocytes (Foster et al., 2013). This further supports the premise that T2Rs can signal through various G proteins. While there is no clear consensus on the precise mechanism, there is an agreement that bitter ligands mediate contractile responses in the vasculature. One study demonstrated a transient drop in blood pressure upon intravenous injection of denatonium benzoate into rats (Lund et al., 2013). Additionally, Manson et al. (2014) attributed the endothelium-independent relaxation of precontracted human pulmonary arteries to the application of bitter ligands for T2Rs (3, 4, 10, and 14). In contrast, denatonium benzoate has been shown to enhance the tone of endothelium-denuded rat aorta rings, which was attributed to specific Tas2r activation (Tas2r40, 108, 126, 135, 137, 143) via Gα_Gustducin_ (Liu et al., 2020). Whether the actions of T2Rs in cardiomyocytes have a direct effect on the force and strength of contraction of individual myocytes remains to be determined. Equally, there is a possibility that these receptors may be expressed in other cell populations, including the specific cells of the conduction system (SA node, AV node, Purkinje Fibers).

## Naturally Occurring Polymorphisms and Disease

GPCRs and their respective ligands have profound homeostatic and regulatory effects on the cardiovascular system. Not surprisingly, mutations and modifications of cardiovascular GPCRs, G proteins and their regulatory proteins are linked to dysfunction and disease (Foster et al., 2015b). T2Rs are one of the most heterogenous and unique families of GPCRs and are now considered as a separate group of receptors (Di Pizio and Niv, 2015). According to the HGNC database, there are 39 genetically diverse and highly polymorphic *TAS2R* single exon genes that encode for 29 functional T2Rs (and 10 non-coding pseudogenes) in humans (Devillier et al., 2015). This is in contrast to the majority of literature that cite the existence of only 25 functional T2Rs (Meyerhof et al., 2010; Lossow et al., 2016). On average, *TAS2R* genes contain four single nucleotide polymorphisms (SNPs) of which the vast majority are non-synonyms mutations that encode amino acid substitutions (Kim et al., 2005). Table 2 outlines all of the non-synonymous SNPs present within the population and their penetrance. These *TAS2R* genes are located on chromosomes 5, 7, and 12 (Adler et al., 2000; Foster et al., 2015a), with dense clustering on chromosomes 7 and 12. The close proximity is thought to underpin the enormous variation and diversification of the T2R repertoire within humans.

**TABLE 2:** List of polymorphisms in human cardiac-expressed *TAS2Rs* (penetrance > 1% in the population) sourced from UCSC Genome Browser and NCBI SNP databases.

	**Name**	**Penetrance (>1%)**	**Location**	**rs number**	**Average heterozygosity**	**Literature phenotype link**
T2R3	–	–	–	–	–	
T2R4	F7S	F (T**T**C) - 52.69%; S (T**C**C) - 47.31%	TM1	rs2233998	0.499 ± 0.025	
	V96L	V (**G**TC) - 48.29%; L (**C**TC) - 51.71%	TM2	rs2234001	0.499 ± 0.017	Sensitivity to stevioside (Risso et al., 2014), coffee consumption (Hayes et al., 2011)
	S171N	S (A**G**T) - 49.43%; N (A**A**T) - 50.56%	TM5	rs2234002	0.500 ± 0.006	
T2R5	S26I	S(A**G**C) - 51.96%; I (A**T**C) - 48.03%	TM1	rs2227264	0.499 ± 0.019	Alcohol consumption (Choi et al., 2017), coffee consumption (Hayes et al., 2011)
T2R9	V187A	V (G**T**G) - 40.62%; A (G**C**G) - 59.38	TM5	rs3741845	0.481 ± 0.095	Glucose homeostasis (Dotson, 2008)
	A233T	A (**G**CA) - 98.99%; T (**A**CA) - 1.01%	TM6	rs77609577	0.019 ± 0.097	
	L304F	L (**C**TT) - 99%; F (**T**TT) - 1%	C terminal	rs3944035	0.02 ± 0.098	
T2R10	T156M	T (A**C**G) - 2.28%; M (A**T**G) - 97.71%	ECL2	rs597468	0.042 ± 0.139	
T2R13	N259S	N (A**A**C) - 41.73%; S (A**G**C) - 58.27%	TM7	rs1015443	0.485 ± 0.084	Alcohol consumption (Dotson et al., 2012), chronic rhinosinusitis (Mfuna Endam et al., 2014)
T2R14	T86A	T (**A**CT) - 98.30%; A (**G**CT) - 1.7%	TM3	rs16925868	0.033 ± 0.125	
	L201F	L (**C**TC) - 98.47%; F (**T**TC) - 1.53%	TM5	rs35804287	0.031 ± 0.121	
T2R16	N172K	N (AA**T**) - 97.15%; K (AA**G**) - 2.85%	TM5	rs846664	0.051 ± 0.151	Sensitivity to cyanogenic glycosides (Soranzo et al., 2005), alcohol dependence (Hinrichs et al., 2006), sensitivity to salicin (Campbell et al., 2014)
	R222H	R (C**G**C) - 31.03%; H (C**A**C) - 68.97%	TM6	rs860170	0.429 ± 0.174	Sensitivity to salicin (Risso et al., 2017)
T2R19	V32I	V (**G**TC) - 98.75%; I (**A**TC) - 1.25%	TM1	rs56985810	0.023 ± 0.104	
	K109T	K (A**A**G) - 98.96%; T (A**C**G) - 1.04%	TM3	rs115193179	0.021 ± 0.101	
	K126Q	K (**A**AG) - 94.9%; Q (**C**AG) - 5.1%	TM4	rs12424373	0.096 ± 0.197	
	R152S	R (AG**A**) - 1.52%; S (AG**T**) - 98.48%	TM4	rs75356565	0.03 ± 0.119	
	I225V	I (**A**TA) - 98.48%; V (**G**TA) - 1.52%	TM6	rs115299813	0.03 ± 0.119	
	K258N	K (AA**A**) - 5.9%; N (AA**T**) - 94.1%	TM7	rs76455106	0.111 ± 0.208	
	L261F	L (**C**TC) - 93.9%; F (**T**TC) - 6.1%	TM7	rs74992161	0.114 ± 0.21	
	C264Y	C (T**G**C) - 91.57%; Y (T**A**C) - 8.43%	TM7	rs76970958	0.154 ± 0.231	
	V267L	V (**G**TT) - 9.36%; L (**C**TT) - 90.64%	TM7	rs74772077	0.177 ± 0.239	
	G282R	G (**G**GA) - 78.67%; R (**A**GA) - 21.33%	TM7	rs72475481	0.336 ± 0.235	
	F290S	F (T**T**T) - 89.88%; S (T**C**T) - 10.12%	Helix 8	rs72475480	0.182 ± 0.241	
	W295C	W (TG**G**) - 93.77%; C (TG**T**) - 5.14%	Helix 8	rs77837442	0.122 ± 0.219	
	W295*	W (TG**G**) - 93.77%; * (TG**A**) - 1.09%	Helix 8	rs77837442	0.122 ± 0.219	
	M297V	M (**A**TG) - 94.4%; V (**G**TG) - 5.6%	C terminal	rs74386164	0.106 ± 0.204	
	R299C	R (**C**GC) - 53.93%; C (**T**GC) - 46.07%	C terminal	rs10772420	0.498 ± 0.035	Grapefruit juice consumption (Hayes et al., 2011), sensitivity to quinine (Reed et al., 2010)
	*300W	* (TG**A**) - 94.12%; W (TG**G**) - 5.88%	Stop codon	rs79475879	0.111 ± 0.208	
T2R20	K79E	K (**A**AA) - 77.44%; E (**G**AA) - 22.56%	TM3	rs7135018	0.350 ± 0.229	
	V141I	V (**G**TT) - 81.6%; I (**A**TT) - 18.4%	TM4	rs79420812	0.300 ± 0.245	
	H143Q	H (CA**C**) - 58.83%; Q (CA**A**) - 41.17%	TM4	rs12226920	0.484 ± 0.087	Chronic rhinosinusitis (Mfuna Endam et al., 2014)
	H148N	H (**C**AC) - 58.64%; N (**A**AC) - 41.36%	TM4	rs12226919	0.485 ± 0.086	Chronic rhinosinusitis (Mfuna Endam et al., 2014)
	I236V	I (**A**TA) - 58.8%; L (**C**TA) - 41.2%	TM6	rs10845281	0.484 ± 0.087	
	F252S	F (T**T**T) - 58.84%; S (T**C**T) - 41.16%	ECL3	rs10845280	0.484 ± 0.087	
	R255L	R (C**G**A) - 58.84%; L (C**T**A) - 41.16%	ECL3	rs10845279	0.484 ± 0.087	
T2R30	I199V	I (**A**TC) - 89.2%; V (**G**TC) - 10.8%	TM5	rs77777159	0.202 ± 0.245	
	Q210H	Q (CA**G**) - 1.40%; H (CA**C**) - 98.60%	ICL3	rs200082783	0.028 ± 0.144	
	S220R	S (AG**C**) - 98.1%; R (AG**A**) - 1.9%	TM6	rs201738458	0.04 ± 0.135	
	F252L	F (TT**T**) - 56.44%; L (TT**G**) - 43.56%	ECL3	rs2599404	0.493 ± 0.06	
T2R31	I33T	I (A**T**T) - 80.43%; T (A**C**T) - 19.57%	TM1	rs763263807	0.315 ± 0.241	
	R35W	R (**C**GG) - 54.73%; W (**T**GG) - 45.27%	TM1	rs10845295	0.496 ± 0.042	
	L48V	L (**C**TC) - 78.68%; V (**G**TC) - 23.16%	TM2	rs760444623	0.335 ± 0.235	
	F71L	F (TT**T**) - 54.55%; L (TT**G**) - 45.45%	TM2	rs78562467	0.496 ± 0.045	
	Y76C	Y (T**A**T) - 75.88%; C (T**G**T) - 24.12%	ECL1	rs80125932	0.366 ± 0.221	
	V80L	V (**G**TA) - 53.43%; L (**T**TA) - 46.57%	TM3	rs73049074	0.498 ± 0.034	
	V87I	V (**G**TC) - 52.73%; I (**A**TC) - 47.27%	TM3	rs73049072	0.499 ± 0.027	
	L98P	L (C**T**T) - 52.29%; P (C**C**T) - 47.71%	TM3	rs73049067	0.499 ± 0.023	
	H120R	H (C**A**C) - 52.34%; R (C**G**C) - 47.66%	ICL2	rs72475488	0.499 ± 0.023	
	M132V	M (**A**TG) - 75.88%; V (**G**TG) - 24.12%	TM4	rs78152338	0.366 ± 0.221	
	Q143H	Q (CA**A**) - 88.86%; H (CA**T**) - 10.97%	TM4	rs115707514	0.206 ± 0.247	
	I147V	I (**A**TA) - 89.57%; V (**G**TA) - 10.43%	TM4	rs199736450	0.187 ± 0.242	
	K150N	K (AA**A**) - 94.91%; N (AA**T**) - 5.1%	ECL2	rs774681705	0.097 ± 0.197	
	E151Q	E (**G**AG) - 94.88%; Q (**C**AG) - 5.12%	ECL2	rs761846423	0.097 ± 0.198	
	R154W	R (**C**GG) - 94.52%; W (**T**GG) - 5.48%	ECL2	rs372544509	0.104 ± 0.203	
	L162M	L (**T**TG) - 21.98%; M (**A**TG) - 76.88%	ECL2	rs10743938	0.357 ± 0.238	
	L162V	L (TTG) - 21.98%; V (**G**TG) - 1.15%	ECL2	rs10743938	0.357 ± 0.238	
	S170R	S (**A**GT) - 95.2%; R (**C**GT) - 4.18%	TM5	rs75346915	0.08 ± 0.183	
	V172M	V (**G**TG) - 95.81%; M (**A**TG) - 4.19%	TM5	rs116298721	0.08 ± 0.184	
	A177T	A (**G**CG) - 97.89%; T (**A**CG) - 2.11%	TM5	rs369562584	0.041 ± 0.138	
	Q217E	Q (**C**AA) - 80.55%; E (**G**AA) - 19.45%	ICL3	rs10845294	0.317 ± 0.241	
	A227V	A (G**C**T) - 56.34%; V (G**T**T) - 43.66%	TM6	rs10845293	0.493 ± 0.059	
	L237F	L (TT**A**) - 98.59%; F (TT**T**) - 1.41%	TM6	rs116926686	0.028 ± 0.115	
	V240I	V (**G**TT) - 57.85%; I (**A**TT) - 42.15%	TM6	rs10772423	0.489 ± 0.074	
	P276R	P (C**C**A) - 81.58%; R (C**G**A) - 18.42%	TM7	rs12318612	0.304 ± 0.244	
	W281C	W (TG**G**) - 98.49%; C (TG**T**) - 1.51%	TM7	rs139069360	0.030 ± 0.119	
	R295W	R (**C**GG) - 98.75%; W (**T**GG) - 1.25%	Helix 8	rs199894662	0.026 ± 0.111	
	V297M	V (**G**TG) - 98.7%; M (**A**TG) - 1.3%	Helix 8	rs201730548	0.026 ± 0.110	
T2R39	–	–	–	–	–	
T2R42	Y175F	Y (T**A**T) - 40.01%; F (T**T**T) - 59.99%	TM5	rs35969491	0.479 ± 0.100	
	*S179–*	*S (A****GA****) - 14.29%; – (A–) - 85.71%*	TM5	*rs747949093*	*0.245 ± 0.250*	
	F196S	F (T**T**C) - 40.03%; S (T**C**C) - 59.97%	TM5	rs5020531	0.479 ± 0.100	Regulation of thyroid hormones (Clark et al., 2015)
	W255G	W (**T**GG) - 83.49%; G (**G**GG) - 16.51%	TM6	rs1669413	0.272 ± 0.249	
	C265Y	C (T**G**C) - 23.68%; Y (T**A**C) - 76.32%	TM7	rs1451772	0.362 ± 0.223	
	Q292R	Q (C**A**A) - 23.7%; R (C**G**A) - 76.3%	Helix 8	rs1669412	0.362 ± 0.223	
	N310K	N (AA**C**) - 83.41%; K (AA**A**) - 16.59%	C terminal	rs1669411	0.273 ± 0.249	
	P311A	P (**C**CT) - 83.42%; A (**G**CT) - 16.58%	C terminal	rs1650017	0.273 ± 0.249	
T2R43	W35S	W (T**G**G) - 66.15%; S (T**C**G) - 33.85%	TM1	rs68157013	0.447 ± 0.155	
	L48V	L (**C**TC) - 69.68%; V (**G**TC) - 30.32%	TM2	rs113197337	0.423 ± 0.181	
	DQILTA45-50	DQILTA - 98.39%; - 1.61%	TM2	rs200922417	0.032 ± 0.122	
	N76Y	N (**A**AT) - 93.56%; Y (**T**AT) - 6.44%	ECL1	rs200999522	0.120 ± 0.214	
	V80L	V (**G**TA) - 97.99%; L (**T**TA) - 2.01%	TM3	rs73064968	0.039 ± 0.135	
	*I91T*	*I (A****T****C) - 96.7%; T (A****C****C) - 3.3%*	*TM3*	*rs201085601*	*0.064 ± 0.167*	
	*N92S*	*N (A****A****C) - 96.8%; S (A****G****C) - 3.2%*	*TM3*	*rs199553429*	*0.062 ± 0.165*	
	F116L	F (**T**TT) - 94.52%; L (**C**TT) - 5.48%	ICL2	rs201210705	0.104 ± 0.203	
	H120R	H (C**A**C) - 79.18%; R (C**G**C) - 20.82%	ICL2	rs201460452	0.330 ± 0.237	
	M132V	M (**A**TG) - 70.22%; V (**G**TG) - 29.78%	TM4	rs11526470	0.418 ± 0.185	
	I147V	I (**A**TA) - 60.19%; V (**G**TA) - 39.81%	TM4	rs73064966	0.479 ± 0.100	
	E151Q	E (**G**AG) - 96.38%; Q (**C**AG) - 3.62%	ECL2	rs201455884	0.070 ± 0.173	
	R154G	R (**C**GG) - 98.01%; G (**G**GG) - 1.9%	ECL2	rs200586631	0.038 ± 0.133	
	K169R	K (A**A**G) - 98.25%; R (A**G**G) - 1.75%	TM5	rs201365712	0.034 ± 0.127	
	S170R	S (**A**GT) - 97.53%; R (**C**GT) - 2.47%	TM5	rs200838689	0.048 ± 0.147	
	S170R	S (AG**T**) - 97.67%; R (AG**G**) - 2.33%	TM5	rs116243872	0.046 ± 0.145	
	F174L	F (**T**TT) - 97.11%; L (**C**TT) - 2.89%	TM5	rs113441874	0.056 ± 0.158	
	N176D	N (**A**AT) - 96.1%; D (**G**AT) - 3.9%	TM5	rs200422162	0.075 ± 0.179	
	M177T	M (A**T**G) - 95.6%; T (A**C**G) - 4.4%	TM5	rs114386807	0.084 ± 0.187	
	V182L	V (**G**TA) - 94.49%; L (**C**TA) - 5.51%	TM5	rs72477447	0.104 ± 0.203	
	L190V	L (**C**TG) - 96.64%; V (**G**TG) - 3.36%	TM5	rs200392796	0.068 ± 0.172	
	L193I	L (**C**TA) - 97.01%; I (**A**TA) - 2.99%	TM5	rs202247625	0.058 ± 0.160	
	M196L	M (**A**TG) - 97.38%; L (**C**TG) - 2.62%	TM5	rs200974913	0.051 ± 0.151	
	I199V	I (**A**TC) - 97.78%; V (**G**TC) - 2.22%	TM5	rs78179946	0.043 ± 0.141	
	C200F	C (T**G**T) - 97.91%; F (T**T**T) - 2.09%	TM5	rs144622176	0.041 ± 0.137	
	Q210H	Q (CA**G**) - 88.86%; H (CA**C**) - 10.97%	ICL3	rs201245949	0.091 ± 0.193	
	H212R	H (C**A**T) - 55.77%; R (C**G**T) - 44.23%	ICL3	rs71443637	0.491 ± 0.066	
	A227V	A (G**C**T) - 58.97%; V (G**T**T) - 41.03%	TM6	rs73064964	0.490 ± 0.07	
	L235F	L (**C**TC) - 81.26%; F (**T**TC) - 18.74%	TM6	rs3759244	0.305 ± 0.244	
	C238R	C (**T**GT) - 94.63%; R (**C**GT) - 5.37%	TM6	rs3759245	0.102 ± 0.201	
	*G253R*	*G* (***G****GA) - 92.5%; R* (***A****GA) - 7.5%*	*ECL3*	*rs202114077*	*0.139 ± 0.224*	
	*G253E*	*G (G****G****A) - 92.6%; E (G****A****A) - 7.4%*	*ECL3*	*rs200981579*	*0.137 ± 0.223*	
	*S254N*	*S (A****G****T) - 92.9%; N (A****A****T) - 7.1%*	*ECL3*	*rs201300744*	*0.132 ± 0.220*	
	K265Q	K (**A**AA) - 97.46%; Q (**C**AA) - 2.54%	TM7	rs200291442	0.050 ± 0.149	
	R268G	R (**A**GA) - 96.89%; G (**G**GA) - 3.11%	TM7	rs202101405	0.060 ± 0.163	
	Y271C	Y (T**A**T) - 96.54%; C (T**G**T) - 3.46%	TM7	rs201618803	0.067 ± 0.170	
	P272S	P (**C**CT) - 96.53%; S (**T**CT) - 3.47%	TM7	rs200533679	0.067 ± 0.170	
	I274V	I (**A**TC) - 96.44%; V (**G**TC) - 3.56%	TM7	rs201681140	0.069 ± 0.172	
	I274T	I (A**T**C) - 96.32%; T (A**C**C) - 3.68%	TM7	rs200479139	0.071 ± 0.174	
	F290Y	F (T**T**T) - 83.84%; Y (T**A**T) - 16.16 %	Helix 8	rs111846092	0.282 ± 0.248	
	F294L	F (TT**T**) - 84.25%; L (TT**G**) - 15.75%	Helix 8	rs73064960	0.276 ± 0.249	
	W300*	W (TG**G**) - 90.86%; * (TG**A**) - 9.14%	C terminus	rs3759247	0.166 ± 0.236	
T2R45	–	–	–	–	–	
T2R46	*T16I*	*T (A****C****A) - 98.25%; I (A****T****A) - 1.75%*	*TM1*	*rs201410559*	*0.034 ± 0.127*	
	V61G	V (G**T**C) - 98.64%; G (G**G**C) - 1.36%	TM2	rs201585352	0.027 ± 0.113	
	W60–	W (**T**GG) - 98.64%; – (-GG) - 1.36%	TM2	rs201847607	0.027 ± 0.113	
	I132M	I (AT**A**) - 98.89%; M (AT**G**) - 1.11%	TM4	rs770484573	0.022 ± 0.103	
	V141A	V (G**T**T) - 66.73%; A (G**C**T) - 33.27%	TM4	rs200936852	0.444 ± 0.158	
	I147V	I (**A**TA) - 58.3%; V (**G**TA) - 41.7%	TM4	rs72477411	0.486 ± 0.082	
	I153V	I (**A**TA) - 55.36%; V (**G**TA) - 44.64%	ECL2	rs72477410	0.494 ± 0.053	
	S170R	S (**A**GT) - 77.29%; R (**C**GT) - 22.71%	TM5	rs200171449	0.351 ± 0.229	
	N176D	N (**A**AT) - 97.86%; D (**G**AT) - 2.14%	TM5	rs766258006	0.042 ± 0.138	
	I181M	I (AT**C**) - 97.99%; M (AT**G**) - 2.01%	TM5	rs748842122	0.039 ± 0.135	
	L190V	L (**C**TG) - 98.57%; V (**G**TG) - 1.43%	TM5	rs779108518	0.028 ± 0.115	
	L228M	L (**T**TG) - 57.8%; M (**A**TG) - 42.2%	TM6	rs2708380	0.489 ± 0.074	
	W250*	W (T**G**G) - 77.43%; * (T**A**G) - 22.57%	TM6	rs2708381	0.350 ± 0.229	
T2R50	C203Y	C (T**G**T) - 60.27%; Y (T**A**T) - 39.73%	TM5	rs1376251	0.479 ± 0.101	Myocardial infarction (Shiffman et al., 2005, 2008; Tsygankova et al., 2017), coronary heart disease (Yan et al., 2009)

Italicized polymorphisms represent those that have less than 300 alleles detected in the sample population (* denotes stop codon).

The importance of uncovering the primary function of T2Rs in the heart is supported by the critical role they play within the respiratory system. *TAS2R*38 is expressed in all aspects of the upper and lower respiratory tracts including sinonasal epithelial cells, bronchial epithelial cells, bronchial smooth muscle, and pulmonary vasculature smooth muscle (Shah et al., 2009; Grassin-Delyle et al., 2013; Upadhyaya et al., 2014; Devillier et al., 2015). Application of phenylthiocarbamide (PTC) or two quorum sensing molecules (C4HSL and C12HSL) secreted by *Pseudomonas aeruginosa* were shown to increase mucociliary clearance, bronchodilation, and production of bactericidal levels nitric oxide in explanted human tissue samples and primary airway–liquid interface cultures (Lee et al., 2012). This supports the recent finding that T2Rs play a role in innate immunity, as quorum sensing molecules serve to communicate between bacterial populations, allowing them to establish themselves during infection (Lee et al., 2014). Bitter taste receptors, particularly *TAS2R*38, are a unique and diverse family of GPCRs due to the number of their naturally occurring genetic variants (Kim et al., 2005). Compared to the functional (PAV) haplotype, individuals with the non-functional (AVI) haplotype were shown to be more susceptible to respiratory infections as the receptor was unable to detect the compounds and respond appropriately (Lee et al., 2012). A similar result was seen with regard to oral innate immunity (Gil et al., 2015). *TAS2R*38 PAV/PAV mRNA was upregulated ∼4.3-fold in response to *Streptococcus mutans* bacteria (over the unstimulated control) whereas the AVI/AVI was only ∼1.2-fold. Furthermore, the level of hBD-2 (antimicrobial peptide) induced was highest in those with the PAV/PAV genotype (Gil et al., 2015). On this basis, the authors concluded that a person’s T2R38 genotype determines oral innate immunity.

Natural polymorphisms are no longer thought only to account for differences in oral bitter taste perception (Roudnitzky et al., 2016). It is now recognized that these polymorphisms also influence other important aspects of our physiology including alcohol dependence, eating behavior, longevity, glucose homeostasis and regulation of thyroid hormones (Dotson, 2008; Hayes et al., 2011; Campa et al., 2012; Clark et al., 2015). There are 132 naturally occurring non-synonymous polymorphisms for cardiac-expressed T2Rs and it is clear that the majority of these remain uncharacterized (Table 3). One polymorphism that is of particular interest is T2R50-rs1376251, as debate remains in the literature over its potential association with myocardial infarction and coronary heart disease (Shiffman et al., 2008; Tepper et al., 2008; Yan et al., 2009; Koch et al., 2011; Ivanova et al., 2017; Tsygankova et al., 2017). There are also polymorphisms outside of the taste receptor coding region, or those that result in synonymous mutations that have been associated with changes in physiology. Of note, T2R14 rs3741843 has been associated with decreased sperm motility (Gentiluomo et al., 2017). Individuals that were homozygous carriers for the (G) allele, encoding arginine (R – AGG), showed a decreased sperm progressive motility compared to heterozygotes and homozygotes for the (A) allele, which encodes arginine (R – AGA). The authors rationalized using *in silico* analysis that T2R14 regulates the expression of T2R43. Furthermore, an upstream mutation of *TAS2R*3 rs11763979 can regulate the expression of WEE2 antisense RNA one (WEE2-AS1), which increases the expression of WEE2 within the testis. WEE2 is a protein tyrosine kinase involved in the regulation of cell cycle progression (Nakanishi et al., 2000). Overexpression of WEE2 in the testis was hypothesized to increase the number of abnormal sperm cells (Gentiluomo et al., 2017). Despite recent progress, it is unclear the full extent to which polymorphisms can influence T2R physiology, although it is clear investigation into their effects is warranted.

**TABLE 3:** List of ligands known to activate cardiac specific T2Rs and their classification, number of T2Rs activated [bold indicates the receptor corresponding to the lowest threshold (TC) or effective concentration (EC50) *in vitro* concentration], reported effects in the cardiovascular system and corresponding dose/serum (* = based on 5.5 L of blood in human body, or without First Pass Effect of liver).

	**Classification**	**Activates T2R** (Jaggupilli et al., 2016)	***In vitro* (μM) - TC/EC50** Jaggupilli et al. (2016)	**Reported effects on cardiovascular system**	**Reported doses**	**Reported or equivalent serum (μM)**
**Bitter compounds in food**						
Absinthin	Sesquiterpene lactone	10, 14, **30**, 46	0.4 ± 0.06 (EC50)	–	–	–
Acesulfame K	Artificial sweetener	**31**, 43	2500 ± 10 (EC50)	–	–	–
Apigenin	Flavonoid	**14**, 39	20.5 (EC50)	–	–	–
Amarogentin	Secoiridoid glycoside	30, 39, 43, **46**, 50	65 ± 16 (EC50)	–	–	–
Andrographolide	Diterpenoid lactone	30, **46**, 50	13 ± 2.17 (EC50)	Shortened AP duration and reduced maximum upstroke (rabbits) (Zeng et al., 2017)	–	–
Aristolochic acid	Carcinogen	14, 31, **43**	0.081 ± 0.0008 (EC50)	Valvular heart disease - aortic sufficiency (Vanherweghem, 1997)	–	–
Caffeine	Stimulant	10, 14, 43, 46	300 (TC)	Tachycardia, arrhythmia (Cappelletti et al., 2018)	80–100 mg/L	>400
Datiscetin	Flavonoid	**14**, 39	10 (EC50)	–	–	–
(-)-Epicatechin	Antioxidant	4, 5, **39**	417.7 (EC50)	Promotes vasodilation (increase NO and decrease endothelin-1) (Schroeter et al., 2006; Loke et al., 2008; Mannaerts et al., 2017)	–	–
(-)-Epicatechin gallate (Ecg)	Flavonoid	**14**, 39	70 (EC50)	–	–	–
(-)-Epigallocatechin gallate (EGCg)	Flavonoid	**14**, 39	34 (EC50)	Reduction of diastolic BP (Brown et al., 2009)	800 mg	∼300 (without First Pass Effect)*
Falcarindiol	Antitumorigenic	14	100 (TC)	–	–	–
Genistein	Phytoestrogen	**14**, 39	28.9 (EC50)	–	–	–
Histidine	α-Amino acid	39	430 (TC)	Arrhythmia prevention, inotropic support (Careaga et al., 2001; Teloh et al., 2016)	198 mM	8000–21000*
Humulone isomers	Alpha acid	14	0.01 (TC)	Inhibit VEGF mediated angiogenesis and endothelial proliferation (mouse) (Shimamura et al., 2001)	–	100
Naringenin	Flavonoid	14, **39**	32.9 (EC50)	–	–	–
Procyanidin	Flavonoid	5	35.6 ± 0.7 (EC50)	Improved hemodynamic parameters and collagen content (rats) (Martin-Fernandez et al., 2014)	6500 mg (65 kg human, 100 mg/kg)	∼2000 (without First Pass Effect)*
Quercetin	Flavonoid	14	1 (TC)	Reduction in BP (Serban et al., 2016)	>500 mg	∼300 (without First Pass Effect)*
Sodium benzoate	Preservative	**14**, 16	300 (TC)	Caffeine alkaloid - combined with caffeine (Yucel et al., 1999)	–	–
Sinigrin	Glucosinolate	16	100 (TC)	–	–	–
Thiamine	Vitamin B1	39	1000 (TC)	Deficiency results in wet beriberi (Lei et al., 2018)	–	–
Thujone, (-)-α	Stimulant	10, **14**	15 (EC50)	Arrhythmia, hypotension, vasodilation (rats) (Pinto-Scognamiglio, 1968)	12480 mg (65 kg human, 192 mg/kg)	15000 (without First Pass Effect)*
**Chemicals/drugs**						
Allylthiourea	Nitrification inhibitor	50	720 ± 150 (EC50)	–	–	–
Atropine	Muscarinic antagonist	10, 46	100 (TC)	Tachycardia, arrhythmogenic (mice) (Perera et al., 2017)	–	–
Azathioprine	Immuno- suppressant	4, 10, **14**, 46	100 (TC)	Atrial fibrillation, hypotension, tachycardia, cardiogenic shock (Dodd et al., 1985; Brown et al., 1997)	50 mg/day (2 weeks before hospital admission)	32 (without First Pass Effect)*
Azithromycin	Antibiotic	4	74.45 ± 12.3(EC50)	Ventricular tachycardia, prolongation of QT interval, torsades de pointes (Russo et al., 2006; Trifiro et al., 2017)	500 mg/day i.v.	∼115*
4,4-Bipyridine	Bipyridine	**10**, 14, 16	3680 ± 60 (EC50)	Other bipyridines used in heart disease and cardiac arrhythmias	–	–
Benzamide	Benzamides	14	300 (TC)	Substituted benzamides linked to hypotension, prolongation of QT interval, ventricular arrhythmias	–	–
Carisoprodol	Muscle relaxer	14	100 (TC)	Tachycardia, hypotension, heart palpitations (Rohatgi et al., 2005; Vo et al., 2017)	71 mg/L	>250
Chloroquine	Antimalarial	**3**, 10, 39	172 ± 29 (EC50)	Cardiomyopathy, hypertrophy, ventricular arrhythmias: ST-segment depression, T wave inversion and QT interval prolongation, relaxation (Edwards et al., 1978; Stas et al., 2008; Tonnesmann et al., 2013)	300 mg	>900 (without First Pass Effect)*
Chlorpheniramine	Antihistamine	4, **10**, 14, 39, 46	10 (TC)	QT interval prolongation, torsades de pointes tachycardia (Nia et al., 2010)	2.5 mg (2.5 mg, two capsules three time a day)	∼10 (without First Pass Effect)*
Chloramphenicol	Antibiotic	10, 39, 41, 43, **46**	10 (TC)	Gray Baby Syndrome - hypotension, arrhythmias; cardiovascular collapse (Sutherland, 1959; Biancaniello et al., 1981; Suarez and Ow, 1992)	313 mg/L	>950
Clonixin	NSAID	14	2 (TC)	Cardiodepression and hypotension (rats) (Bustamante et al., 1989; Morales et al., 1995)	7800 mg (65 kg human, 120 mg/kg - lethal dose i.v. rats)	>5000 (without First Pass Effect)*
Chlorhexidine	Antiseptic	14	0.1 (TC)	Hemodynamic instability and vasodilatory shock (Guleri et al., 2012; Zhou et al., 2019)	Chlorhexidine-coated central venous catheter	–
Colchicine	Antigout	4, 39, 46	1025 ± 121 (EC50)	Decreased rates of atrial fibrillation, pericarditis and atherosclerotic vascular disease; cardiac arrhythmias and cardiovascular collapse (Macleod and Phillips, 1947; Papageorgiou et al., 2017; Thompson, 2019)	1 mg/day	0.45 (without First Pass Effect)*
Cycloheximide	Eukaryote protein synthesis inhibitor	10	100 (TC)	–	–	–
Cromolyn	Mast cell stabilizer	**20**, 43	42 ± 25 (EC50)	Attenuates adverse LV remodeling and dysfunction in myocarditis, restored cardiac contractile dysfunction (rats) (Santone et al., 2008; Mina et al., 2013)	1625 mg (65 kg human, 25 mg/kg i.p. rats)	630 (without First Pass Effect)*
Dapsone	Antibiotic	4, 10	100 (TC)	Myocardial injury, shock, ventricular dysrhythmia, cardiac arrest. Hypertension (Kang et al., 2016) (Lau, 1995; Zhu et al., 2009)	300 mg–3 g	219–2190 (without First Pass Effect)*
Denatonium benzoate	Deterring agent	4, 10, 13, **30**, 39, 43, 46	0.27 ± 0.06 (EC50)	Vasodilation (rats) (Lund et al., 2013)	1 μM i.v. in rats	–
Dextromethorphan	Sedative	10	10 (TC)	QT interval prolongation, torsades de pointes tachycardia, hypertension (Kaplan et al., 2011; Wu et al., 2012; Upadhyaya et al., 2014; Chew et al., 2017)	1920 mg (27 mg/kg)	>1000 (without First Pass Effect)*
Diphenhydramine	Antihistamine	14	30 (TC)	QT interval prolongation, ventricular tachycardia, hemodynamic collapse, cardiac arrest, junctional rhythm, complete right bundle branch block, hypotension (Yu et al., 2016; Abernathy et al., 2017; Labarinas et al., 2018; Nishino et al., 2018)	18.7 mg/L	73
Diphenidol	Antiemetic	4, 10, 13, 14, 16, 20, 30, **31**, 39, 43, 46	3 (TC)	Contraction band necrosis (post mortem), hypotension, arrhythmia, including QT interval prolongation, T wave change, U wave appearance, AV block, bundle branch block, ventricular premature contraction, ventricular tachycardia and ventricular fibrillation (Wasserman et al., 1975; Yang and Deng, 1998; Zhang et al., 2015)	45 mg/L	145
Erythromycin	Antibiotic	10	300 (TC)	QT interval prolongation, torsades de pointes tachycardia, 68% increased of hospital-acquired cardiac events (arrhythmia, heart failure, myocardial ischemia) (Giudicessi et al., 2018; Postma et al., 2019)	1300 mg (65 kg human, 15–20 mg/kg i.v. every 6 h)	>300*
Ethylhydrocupreine	Antibiotic	14	10 (TC)	–	–	–
Famotidine	Antacid	10, 31	300 (TC)	Cardiac arrest, third degree heart block, decreased stroke volume and cardiac output (Kirch et al., 1989; Schoenwald et al., 1999; Lee et al., 2004)	2x 20 mg i.v. dose	>20*
Flufenamic acid	NSAID	14	0.137 ± 0.017 (EC50)	Hypertension and congestive heart failure (Miyamori et al., 1985)	600 mg	>350 (without First Pass Effect)*
Haloperidol	Antipsychotic	10	30 (TC)	Prolongation of QT interval, torsades de pointes, sudden cardiac death (Meyer-Massetti et al., 2010; Fernandes et al., 2018; Vesely et al., 2019)	2–1540 mg I.v. dose (cumulative)	0.9–740*
Hydrocortisone	Medication form of cortisol	46	30 (TC)			
Levofloxacin	Antibiotic	4, **14**, 20	74.69 ± 20.5(EC50)	Ventricular tachycardia, prolongation of QT interval, torsades de pointes (Basyigit et al., 2005; Lu et al., 2015; Okeahialam, 2015)	500 mg/day i.v.	∼250*
Ofloxacin	Antibiotic	9	200 (EC50)	–	–	–
Orphenadrine	Anticholinergic/antihistamine	46	30 (TC)	Prolongation of QT interval, torsades de pointes, bradycardia, asystole (Malizia et al., 1980; Danze and Langdorf, 1991; Luzza et al., 2006)	16.2 mg/L	>60
Methoxsalen/Xanthotoxin	Small molecule - inhibits DNA synthesis	10, **14**, 20	10	–	–	–
Noscapine	Antitussive	14	10 (TC)	Hypotension, relaxation (Weaver et al., 1958; Manson et al., 2014)	–	100
Parthenolide	Antispasmodic	10, 31, **46**	1 (TC)	–	–	–
Pentagalloyl glucose (PGG)	Antitumorigenic	5, **39**	6.6 (EC50)	Modulates perivascular inflammation and prevents vascular dysfunction in Ang II-induced hypertension (mice) (Mikolajczyk et al., 2019)	650 mg (65 kg human, 10 mg/kg i.p.)	∼125 (without First Pass Effect)*
1, 10-Phenanthroline	Antimicrobial	5	100 (TC)	–	–	–
Picrotoxinin	Stimulant	10, 14, 30, **46**	18 (EC50)	AV block, ventricular premature contraction and/or ventricular tachycardia (rats) (Lee et al., 1972, 1974; Lin et al., 1992)	1300 mg (65 kg human, 20 mg/kg i.v.)	∼12*
Pirenzepine	Anticholinergic	9	1800 (EC50)	Increased heart rate turbulence, augmented baroreceptor reflex sensitivity (Pedretti et al., 1995; Vukajlovic et al., 2006)	50 mg/day	∼25 (without First Pass Effect)*
Procainamide	Antiarrhythmic	9	2800 (EC50)	Decrease contractility, hypertension, cardiovascular depression and collapse, prolonged PR/QT intervals and QRS complex, AV block, asystole, bundle branch block, ventricular premature contraction, ventricular tachycardia and ventricular fibrillation (Perkins and Marill, 2012; Ortiz et al., 2017; Osadchii, 2018)	1300 mg (65 kg human, 10 mg/kg/20 min (i.v.)	∼1000*
Quinine	Antimalarial	4, **10**, **14**, **31**, **39**, **43**, **46**	10 (TC)	Hypotension, prolonged PR/QT intervals and QRS complex, bundle branch block, ventricular premature contraction, ventricular tachycardia and ventricular fibrillation (Danopoulos et al., 1954; Ortiz et al., 2017; Osadchii, 2018)	10 mg/L	∼31
Salicin	Anti-inflammatory	16	1400 ± 200 (EC50)	–	–	–
Salicylic acid	Derivative of aspirin	14	1000 (TC)	Supraventricular tachycardia, prolonged asystole, atrial fibrillation (Mukerji et al., 1986; Ihama et al., 2007)	0.49–1.1 mg/mL	∼3500–8000
Strychnine	Pesticide	10, **46**	0.43 ± 0.02 (EC50)	Cardiac arrest, bradycardia, ECG changes (Heiser et al., 1992; Wood et al., 2002; Ponraj et al., 2017)	3.8 mg/L	∼11
2-Thiouracil	Antithyroid	4, **14**, 46	100 ± 10 (EC50)	–	–	–
Tobramycin	Antibiotic	14, **20**	50.97 ± 19.37 (EC50)	Cardiodepression, hypotension, decreased CO, ventricular contractile force (dogs) (Adams et al., 1979)	1300 mg (65 kg human, 20 mg/kg i.v.)	∼500*
Yohimbine	Erectile dysfunction	4, 10, 46	300 (TC)	Promote cardiac noradrenaline release (mice) (Wang et al., 2013)	260 mg (65 kg human, 4 mg/kg)	>130 (without First Pass Effect)*
**Endogenous factors**						
Alanine	α-Amino acid	39	580 ± 10 (EC50)	–	–	–
Androsterone	Steroid hormone	46	1 (TC)	–	–	–
Pantothenic acid	Vitamin B5	14, 31, 43	1000 (TC)	–	–	–
Progesterone	Steroid hormone	46	3 (TC)	QTc shortening (double autonomic blockade, atropine and propranolol) - opposite effect of estradiol (Barbagallo et al., 2001; Yang et al., 2010; Salem et al., 2016; Barber et al., 2019)	6500 μg (65 kg human, 100 μg/kg i.v.)	>3.5*
Taurocholic acid	Primary bile acid	4	300 (TC)	Afterdepolarizations, atrial fibrillation, prolongation of contractile refractory period (Desai and Penny, 2013; Rainer et al., 2013)	–	300–1000
**Bacterial toxins/metabolic by-products**						
Equol	Nonsteroid estrogen	14, 39	100	–	–	–
4-Hydroxy-2-heptylquinolone (HHQ)	*Pseudomonas aeruginosa* quinolone	14	100	–	–	–
Homoserine lactone, *N*-butyryl-L*-*	Bacterial quorum sensing	14	50	–	–	–
Homoserine lactone, *N*-hexanoyl-L-	Bacterial quorum sensing	10	2400 (TC)	–	–	–
Homoserine lactone, *N*-octanoyl-L-	Bacterial quorum sensing	4, **14**, 20	20 ± 10 (EC50)	–	–	–
Homoserine lactone, *N*-3-oxooctanoyl-L-	Bacterial quorum sensing	**4**, 10, 14, 20	41 ± 13 (EC50)	Bradycardia (rats) (Gardiner et al., 2001)	650 mg (65 kg human, 10 mg/kg i.v.)	>450*
*Pseudomonas* quinolone signal (PQS)	*Pseudomonas aeruginosa* quinolone	**4**, 16, 39	100	–	–	150 (Morales-Soto et al., 2018)

## Potential Cardiovascular T2R Ligands

T2Rs are unique as they lack most of the conserved motifs of the class A GPCR family (Lagerstrom and Schioth, 2008). The intracellular loops – regions necessary for signal transduction and feedback modulation (Moreira, 2014), were shown to be more conserved across T2Rs than the extracellular loops that are generally implicated in receptor binding (Meyerhof, 2005). Using T2R14 as an example, Nowak et al. (2018) demonstrated that *in vitro* mutagenesis of 19 receptor mutants (all within the binding pocket) retained the ability to bind at least one of the 7 tested agonists while some improved signaling compared to the wild type. These results are consistent with previous literature that ligands bind within the transmembrane and extracellular domain regions (Brockhoff et al., 2010; Upadhyaya et al., 2015). Interestingly, of the highly expressed cardiac T2Rs, T2R10, T2R14, and T2R46 were shown to bind a wide array of ligands, which is considered disproportional in comparison to the others (Meyerhof et al., 2010). Over 75% of the list of ligands in Table 3 were shown to activate these three broadly tuned T2Rs.

Universally, researchers have used chemicals that ‘taste bitter’ to test for potential ligands. However, if heart tissue expresses over half of the T2Rs family, a major question arises - what is the source of ligands for these T2Rs within the cardiovascular system? We would argue there are four major sources: (1) bitter compounds in food, (2) endogenously produced factors, (3) bacterial metabolic by-products and toxins and (4) chemicals/drugs (outlined in Table 3).

The post-prandial concentration of bitter compounds in the blood increases. One perhaps common example of this is caffeine, which reportedly modulates calcium signaling via interaction with the ryanodine receptor (Kong et al., 2008). Interestingly, caffeine also activates T2R10, -14, and -46 (Meyerhof et al., 2010; Cappelletti et al., 2018) at concentrations that occur in blood post-prandially and which are equivalent to levels that modulate the ryanodine receptor (Kong et al., 2008). In the gut, caffeine activation of T2R has been linked to gastric secretion (Liszt et al., 2017). Caffeine may also act as a stimulate for the central nervous system via the antagonism of adenosine receptors (Fisone et al., 2004). Hence in considering the homeostatic consequence of bitter compounds (such as caffeine) one must also accept that at high concentrations they are interacting with multiple receptor systems. We would anticipate that many bitter compounds in food would have actions on both T2Rs and other targets.

Another interesting possibility is that the body produces endogenous factors that could activate T2Rs. Currently, alanine, pantothenic acid (vitamin B5), steroids (androsterone and progesterone) and taurocholic acid (primary bile acid) have all been identified as ligands for specific receptors (Ji et al., 2014; Lossow et al., 2016). Potentially, the cardiotonic steroids may be ligands for cardiac-expressed T2Rs, although ouabain has already been shown not to be an agonist *in vitro* (Meyerhof et al., 2010), despite being able to augment calcium transients in arterial smooth muscle (Arnon et al., 2000). The other members of this family could also be investigated as potential ligands for cardiac T2Rs. Whether hormones/factors produced by other tissues, or indeed paracrine factors released from cardiac cells, can bind and activate cardiac T2Rs remains to be determined, but is an area of intense interest.

A more provocative idea is that colonizing bacteria, in complex organisms, could produce bitter compounds, including metabolic by-products and other signaling molecules that alter our physiology via T2Rs. A recent study showed commensal bacteria are enable to synthesize GPCR ligands that mimic human signaling molecules (Cohen et al., 2017). Broad screening for bacterial metabolites that activate GPCRs (Chen et al., 2019; Colosimo et al., 2019) have identified numerous candidates, but unfortunately these screens have not included the taste receptors. Interestingly, an olfactory receptor (Olfr78) has been reported to respond to short chain fatty acids produced by gut bacteria (Pluznick et al., 2013). Olfr78 KO mice had elevated blood pressure when treated with antibiotics. As for the T2Rs, T2R38 although its expression is low in the heart, was shown to be broadly tuned for seven bacterial metabolites (Verbeurgt et al., 2017). It is also worth noting that during infections bacterial toxins could be ‘bitter’ and interact with T2Rs once they reached a certain concentration in the blood. One example is quorum sensing molecules - when they reach a certain concentration, bacteria produce a biofilm in order to evade and survive the host immune defense system (Davies et al., 1998). Therefore, it is plausible that T2Rs may alter cardiovascular physiology in response to systemic infections such as sepsis where dramatic cardiovascular changes are observed, e.g., decreased myocardial contractility, vasodilation, endothelial injury and increased heart rate (Singer et al., 2016).

Finally, it is important to address the possibility that off-target activation of T2Rs may play a role beyond normal physiology and mediate unexpected responses to therapeutic drugs, many of which are bitter. Indeed, the possibility that T2Rs act as the mediators of off-target drug effects due to the prevalence of their expression throughout the body has been discussed previously (Clark et al., 2012). There are numerous drugs/chemicals that have specific, detrimental cardiovascular effects and, moreover, these chemicals have been shown to activate specific T2Rs at concentrations to those that elicit these adverse effects.

## Future Directions

The continuous, proper functioning of the heart is fundamental to life. The discovery of T2Rs expressed in cardiac cells predicts important (but yet to be appreciated) roles in heart physiology, as well as its response to external challenges (e.g., diet, metabolic changes, infections, and drugs). Research and knowledge regarding the physiology of T2Rs within the human heart is challenging, primarily due to the constraints of readily acquiring suitable human heart tissue samples. Furthermore, the lack of homology between rodent Tas2rs and human T2Rs (Foster et al., 2013), limits the utility of gene modified animal models to directly inform human physiology. Additionally, the 29 T2Rs (and their many variants) have been historically difficult to heterologously express on the cell membrane of model cells, and this has impeded further investigation of their signaling properties.

It is important to note, that researchers have ectopically expressed human T2Rs in mice and this has provided strong confirmation that a given ligand (tastants) can activate a specific human T2R (Mueller et al., 2005). Perhaps future experiments might extend this approach to develop transgenic mice expressing human T2Rs in a cell-specific context. Stimulation of these receptors with ligands that selectively bind and activate only human T2Rs could provide important insights into the physiological role(s) of T2Rs in human tissues.

Another critical objective will be to develop appropriate cardiac models that express endogenous receptors and recapitulate cardiac physiology. One major advance in cardiovascular research has been the development of induced pluripotent stem cell-derived human cardiomyocytes (Hudson et al., 2012; Soong et al., 2012) and human cardiac organoids (Nugraha et al., 2019). These models will offer the unique opportunity to modulate T2R expression in cardiomyocytes and to thereby investigate bitter ligand-driven changes in cardiac gene transcription, as well as to define alterations in cardiac contractility and function.

Finally, the ultimate goal will be to attribute T2R-mediated expression, activation and signaling to definitive changes in human cardiovascular function *in vivo*. In order for this to succeed, the following challenges need to be resolved - the promiscuity of bitter receptor–ligand interactions, the elucidation of tissue-specific T2R signaling, as well as the lack of definitive research tools (e.g., selective antibodies to T2Rs and specific receptor antagonists). We anticipate that studies focused on examining the functionality (or lack thereof) for the various highly penetrant, cardiac-expressed T2R polymorphisms may provide the means for unambiguously attributing T2R activation to a specific physiological outcome. Analogous to the advances made with non-functional T2R38 variants (T2R38AVI) in the lung, we predict that non-functional, cardiac-expressed T2Rs can be identified and these will prove to be critical in providing the necessary controls for investigating explanted cardiac tissues.

## Author Contributions

CB, SF, and WT designed the scope and structure of the review. CB collated data. CB, SF, and WT wrote and edited the final manuscript. CB produced the figures and tables in consultation with SF and WT.

## Conflict of Interest

The authors declare that the research was conducted in the absence of any commercial or financial relationships that could be construed as a potential conflict of interest.
